# Structured health care for subjects with diabetic foot ulcers results in a reduction of major amputation rates

**DOI:** 10.1186/1475-2840-12-45

**Published:** 2013-03-13

**Authors:** Matthias Weck, Torsten Slesaczeck, Hartmut Paetzold, Dirk Muench, Thomas Nanning, Georg von Gagern, Andrej Brechow, Ulf Dietrich, Mandy Holfert, Stefan Bornstein, Andreas Barthel, Antje Thomas, Carsta Koehler, Markolf Hanefeld

**Affiliations:** 1 Department of Diabetes, Interdisciplinary Diabetic Foot Unit, Weisseritztal-Kliniken, Freital, Germany; 2 Department of Vascular Surgery, Weisseritztal-Kliniken, Freital, Germany; 3 Department of Interventional Angiology, Weisseritztal-Kliniken, Freital, Germany; 4 Department of Diabetes, Metabolism and Endocrinology, Clinic Bavaria Kreischa, Kreischa, Germany; 5 Technical University Dresden, III Medical Clinic, Dresden, Germany; 6 Endokrinologikum, Bochum, Germany; 7 Center for Clinical Studies, Technical University GWT-TUD GmbH Dresden, Dresden, Germany

## Abstract

**Objective:**

We tested the effects of structured health care for the diabetic foot in one region in Germany aiming to reduce the number of major amputations.

**Research design and methods:**

In a prospective study we investigated patients with diabetic foot in a structured system of outpatient, in-patient and rehabilitative treatment. Subjects were recruited between January 1^st^, 2000 and December 31, 2007. All participants underwent a two-year follow-up. The modified University of Texas Wound Classification System (UT) was the basis for documentation and data analysis. We evaluated numbers of major amputations, rates of ulcer healing and mortality. In order to compare the effect of the structured health care program with usual care in patients with diabetic foot we evaluated the same parameters at another regional hospital without interdisciplinary care of diabetic foot (controls).

**Results:**

684 patients with diabetic foot and 508 controls were investigated. At discharge from hospital 28.3% (structured health care program, SHC) vs. 23.0% (controls) of all ulcers had healed completely. 51.5% (SHC) vs. 49.8% (controls) were in UT grade 1.

Major amputations were performed in 32 subjects of the structured health care program group (4.7%) vs. 110 (21.7%) in controls (p<0.0001). Mortality during hospitalization was 2.5% (SHC) vs. 9.4% in controls (p<0.001).

**Conclusions:**

With the structured health care program we achieved a significant reduction of major amputation rates by more than 75% as compared to standard care.

## Background

Foot ulcers are a major complication in patients with diabetes and remain one of the most common causes for hospitalization and the high costs associated with this disease. Unfortunately, lower extremity amputation (LEA) is a frequent and disabling consequence of diabetes. Major determinants in the pathway to limb loss are peripheral sensomotoric neuropathy, ulceration, infection, and peripheral vascular disease [1-4]. The classification of the diabetic foot considers these factors by grading and staging the diabetic foot wound situation according to the modified University of Texas Wound Classification System (modified UT). The extent of the foot wound is graded by Wagner [5] and the staging of the wound situation is described according to Armstrong et al. [1]. The outcome, especially with respect to LEA, appears to deteriorate with increasing grade and stage of the foot wounds.

It is generally accepted that LEA may potentially be avoided in many patients if a more consequent and aggressive therapeutic regimen would be applied. For example, it has been stated that the deficits in the health care structures related to the treatment of diabetes in Germany and western Europe are especially obvious in patients suffering from diabetic foot. Patients with diabetes experience 30 to 40 times more amputations than people without the disease. Recently the numbers of LEA in Germany were calculated based on current health insurance data. Based on these data, about 20,000 major amputations in subjects with diabetes have been carried out in the year 2003 alone and over the past ten years, the number of amputations per year have not declined in Germany [6]. All this reflects the urgent need to improve the organization of our existing health care structures and there is general consent that the introduction of specific disease management or structured health care programs for diabetic foot may be of great benefit.

The purpose of this study was to establish and validate a structured health care program (SHC) for the diabetic foot introduced in the Southeast of Germany near Dresden compared to standard care of diabetic foot. The primary objective of this structured health care program was to reduce the number of LEA. The modified UT-system served as basis for documentation and data analysis of the diabetic foot SHC program.

## Research design and methods

### Design

First, a contract regulating the organization of a SHC based on integrated outpatient treatment, acute in-patient care and rehabilitative treatment was set up and signed by three large and established institutions responsible for the treatment of diabetic foot in the Dresden region (local branch of Germany’s largest Health Insurance Company (*AOK*), a hospital specialized in acute treatment of diabetic foot (*Weißeritztal-Kliniken Freital-Dippoldiswalde*), and a specialized rehabilitation clinic, (*Clinic BAVARIA Kreischa*).

The inclusion of subjects with diabetes with new foot ulcers was scheduled over a time frame of 8 years with a follow-up investigation period for each individual patient over 2 years.

Patients with diabetic foot were referred to the interdisciplinary diabetic foot ward of the hospital, by general practitioners, specialized diabetes outpatient departments, or other specialists. At the interdisciplinary diabetic foot ward, initial diagnostic procedures were carried out and treatment started. Thereafter, the patients were transferred to the rehabilitation clinic. After discharge of the patients from rehabilitation clinic, a diabetic foot outpatient department carried out semi-annual check-up’s including all necessary further individual interventions over a time frame of two years.

**Figure 1:**
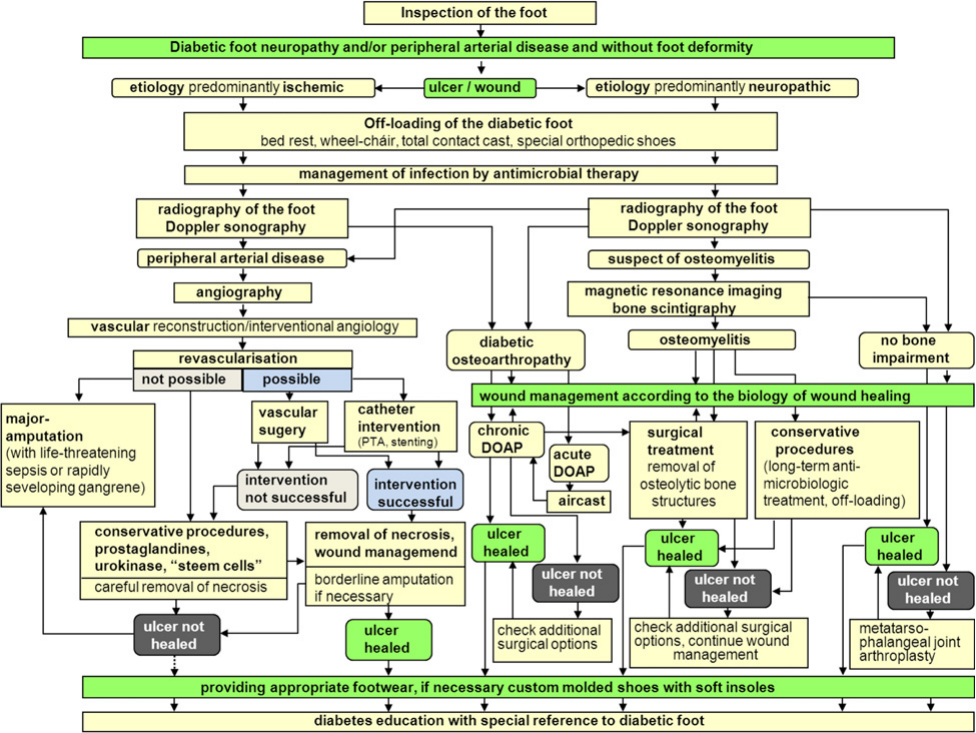
Clinical pathway of diagnosis and treatment of diabetic foot in the structured health care group.

In order to accomplish a standardized clinical procedure, all participating medical institutions shared a common set of diagnostic and therapeutic algorithms (Figure 1). A handbook explaining the standards was available for all professionals involved [7]. All instances were subject to supervision by senior specialists in diabetes (MW, TS, UD).

In Germany there is no central amputation registry or specific data available from Health Insurance Companies. In order to compare the results of the SHC with the usual care of subjects with diabetic foot we recruited a control group at another regional hospital without interdisciplinary care of diabetic foot.

### Study population and procedure

1475 subjects were hospitalized because of diabetic foot ulceration between January 1^st^ 2000 and December 31^st^ 2007. 742 patients out of this group, covered by AOK insurance and presenting with a recently manifested foot ulcer were enrolled consecutively into this observational and prospective study. Exclusion criteria were acute myocardial infarction or stroke within the last 6 months, terminal renal failure or any kind of cancer. Based on these criteria 58 subjects were excluded from the study. All other patients were covered by other Health Insurance Companies. Therefore, 684 subjects with diabetic foot ulceration were suitable for data analysis.

In the control hospital, 560 patients admitted because of diabetic foot were recruited between January 1st 2005 and December 31th 2007. Because of the mentioned criteria 52 subjects were excluded and 508 patients were suitable for data analysis. Health insurance of these subjects was covered by several insurance companies including AOK.

In addition to foot inspection, the physical examination of the patients included the palpation of peripheral pulses and the evaluation of vibration perception threshold with the calibrated Rydell-Seiffer tuning fork at the ankle. Each ulcer was graded and staged using modified UT system.

All patients received identical standard ulcer wound care including use of proper footwear, non-weight bearing limb support, daily wound debridement and careful clinical monitoring. In the case of clinical signs of soft tissue infection or a corresponding antibiogram, antibiotics were prescribed.

The assessment of perfusion included as first line evaluation the palpation of pedal pulses and the measurement of the ankle-brachial index (ABI) using a handheld Doppler device. With this procedure we classified PAD as follows:

Because of false high ABI in subjects with MAC we used continuous wave (cw) Doppler sonography for more detailed analysis of perfusion. We considered compensated perfusion if the increasing wing of the Doppler curve was more steep as the decreasing wing. A gently inclining curve or flat curve were considered as decompensated perfusion or critical limb ischemia.

With these measures we subdivided the patients with diabetic foot into 4 categories:

1. undisturbed perfusion (0.9 < ABI < 1.3)

2. disturbed but compensated perfusion (0.5 < ABI < 0.9)

3. decompensated perfusion and critical limb ischemia (ABI < 0.5)

4. medial arterial calcification (MAC) (ABI > 1.3)

Patients with signs of ischemia (decompensated ischemia) were seen by the interventional angiologist and the vascular surgeon. If vascular reconstruction or interventional radiologic procedures were not possible, prostaglandins, low-dose urokinase or autologous bone marrow derived mononuclear cells (“stem cells”, intramuscular application) were applied in order to improve perfusion [8].

Patients were transferred to the rehabilitation center if acute treatment of the diabetic foot was completed. At the rehabilitation center the treatment procedures of the foot ulcer were continued and combined with an intensified diabetes education program.

The patients were discharged if the diabetic foot wound was healed completely or an outpatient treatment was possible. Before discharge the subjects received definitive individual therapeutic footwear according to the proposals of the German Diabetes Association [9] and the recommendations by Dahmen et al. [10]. The orthopaedic shoes were designed based on measurements of the plantar pressure using the Fastscan in shoe pressure analysis system.

All subjects gave their written informed consent before participating in this study. The study was approved by the local ethics committee.

### Statistical analysis

Data analysis was performed using the IBM SPSS Statistics 19.0 software (SPSS Inc. IBM Company Chicago, US). Kolmogorov-Smirnov test was applied in order to assess normal distribution of each quantitative parameter. Data are expressed as mean ± standard deviation (SD) or as median. Statistical significance was defined as a two-tailed p value of less than 0.05. Differences in means between the groups were tested with the *t*-test for independent samples (normal distribution), Mann–Whitney-U- Test (not normal distribution) or Chi-square-test in categorical variables.

To test the difference of grade and stage of diabetic foot ulcer between the treatment groups at entry into the study we used the *t*-test for independent samples with adjustment of age and PAD. To test the differences of clinical and patient relevant endpoints (mortality, grade and stage of the foot ulcers between both groups at the end of hospital treatment, amputations) we used ANCOVA with adjustment of age, adjustment of grade and stage of the diabetic foot at baseline PAD, history of CHD, hypertension, smoking and MA. For a better quantification of the different grades and stages of diabetic foot in the modified UT classification we implemented a digitalization as follows (Table 1).

**Table 1:** Digitalization of the different grades and stages of diabetic foot ulcers according to the modified UT classification

**Grade and stage of diabetic foot according to the modified UT wound classification system**	**Code**
0A	1
0C	3
1A	11
1B	12
1C	13
1D	14
2A	21
2B	22
2C	23
2D	24
3A	31
3B	32
3C	33
3D	34
4D	44

This means that depending on the extent of grade and stage of diabetic foot disease defined by the modified UT system the numerical value of the codes is increased. Thus, based on this method it is possible to calculate and compare median values for the grade and stage of diabetic foot.

## Results

### Patient structure

684 subjects with newly detected diabetic foot ulcer were consecutively included into the structured health care program and 508 controls were consecutively enrolled into the study. The mean age of the population of the structured health care program was 66.9 ± 10.5 years. Controls were significantly older (71.4 ± 10.8 years; p<0,001).

Diabetes duration (16.1 ± 10.2 vs. 15.8 ± 9.5 years), HbA_1C_ (61.8 ± 14.2 vs. 61.8 ± 14.2 mmol/mol and 7.8 ± 1.8 vs. 7.8 ± 1.8%), BMI (29.7 ± 5.8 vs 29.2 ± 5.7 kg/m^2^) and blood pressure (139 ± 21/76 ±11 vs. 140 ± 25/76 ± 13 mmHg) were comparable between the structured health care program and controls.

With respect to the classification of ulcers (neuroischemic, neuropathic, ischemic), the rate of infection and diabetic complications both groups were comparable.

**Figure 2:**
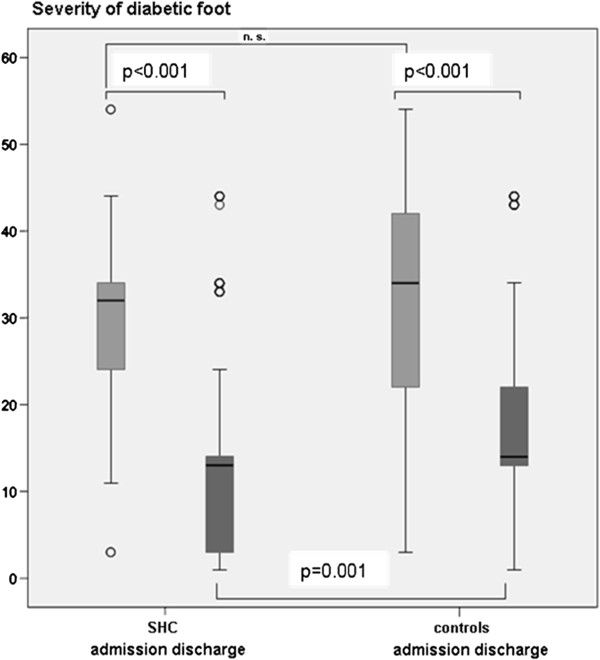
Comparison of ulcer healing rates according to the modified University of Texas wound classification system between subjects with diabetic foot treated by the structured health care program or standard care (controls) test of the structured health care program vs. controls at baseline: *t*-test with adjustment of age; test of change between admission and discharge: ANCOVA with adjustment of age, adjustment of grade and stage of the diabetic foot at baseline, PAD, history of CHD, hypertension, smoking and MA.* The severity of the diabetic foot was calculated according to a coding system described in the text. SHC structured health care program.

The severity of foot ulcers according to the modified UT system was not significantly different between both groups (Figure 2).

The results of the angiological examinations showed that only 7% of the patients of the structured health care program had an undisturbed perfusion (vs. 14.5% controls, p<0.05), 22% had a decompensated perfusion (vs. 15%; p<0.05) and 30% vs. 27% (n. s.) had mediasclerosis.

In the groups of subjects with compensated and decompensated perfusion we performed in the population of the structured health care program 45 infrapopliteal bypass reconstructions (vs. 15 in the controls) and 81 vs. 39 iliacal or femoral arterial reconstructions.

445 vs. 152 patients underwent percutaneous transluminal angioplasty and another 116 patients were treated by prostaglandins, low-dose urokinase, or “stem cells”. 75 controls were treated by prostaglandins.

Detailed data on diabetic complications of both groups are given at Table 2.

**Table 2:** Diabetic complications of the subjects in the structured health care program and controls

**Parameter**	**SHC**	**Controls**
	**n (%)**	**(n%)**
VPT < 4/8	654 (95.6)	457 (90.0)
CAN	289 (42.3)	n. d.
retinopathy		
background	561 (82.0)	n. d.
proliferative	55 (8.0)	n. d.
Microalbuminuria	269 (39.2)	n. d.
creatinine >130μmol/l	104 (15.2)	71 (14.1)
prior amputation	249 (36.4)	
toe	152 (22.2)	n. d.
transmetatarsal	34 (5.0)	n. d.
below the knee	40 (5.8)	73 (14.4)
above the knee	23 (3.4)	53 (10.5)
CAD	567 (82.9)	396 (78.1)
prior AMI	47 (6.9)	41 (8.0)
prior stroke	51 (7.5)	48 (9.5)
hypertension	621 (90.8)	441 (87.0)
dyslipidemia	540 (78.9)	n. d.
smoking	231 (33.8)	158 (31.2)

VPT vibration perception threshold.

CAN cardiac anatomic neuropathy.

CAD coronary artery disease.

SHC structured health care group.

#### Mortality

The mortality rate during the initial hospitalization in the group treated by the structured health care program was 2.5% (n = 17). In contrast the controls had a significantly higher age adjusted mortality rate of 9.4% (n=48, p<0.001). After two years, 419 patients of the structured health care group (63.3% of the original cohort) could still be examined. Altogether, 143 patients (20.9%) died, and 54 (7.9%) dropped out for various reasons and 68 (9.9%) were lost to follow-up. 96 patients in total died in the first year and 47 in the second year of follow-up.

The subjects of the control group had no follow-up examinations.

#### Course of lesions (Table–3)

**Table 3:** Prevalence of wound severity by ulcer grade and stage (modified University of Texas wound classification system) at admission to the clinics (A), discharge from the clinics (B) of the patients with diabetic foot treated by the structured health care program and subjects with diabetic foot treated by standard care (controls) and 2 year follow-up investigation of the structured health care group alone (C) (SHC n= 684, controls n= 508)

**A (admission of patients to the clinic, baseline data)**										
**Modified UT grade (Wagner)**	**0**		**1**		**2**		**3**		**4**	
**UT stage**	**SHC**	**C**	**SHC**	**C**	**SHC**	**C**	**SHC**	**C**	**SHC**	**C**
A n			1	21						
%			0,1	4.1						
B n			19	27	22	34	15	42		
%			2.8	5.3	3.2	6.7	2.2	8.3		
C n	2	41	12	8	34	11	9	2		
%	0.3	8.1	1.8	1.6	5.0	2.2	1.3	0.4		
D n			50	9	199	67	255	109	66	137
%			7.3	1.6	29.1	13.2	37.3	21.5	9.6	26.9
sum n	2	41	82	65	255	112	279	153	66	137
(%)	0.3	8.1	12.0	12.6	37.3	22.1	40.8	30.2	9.6	26.9
**B (discharge of patients from the clinic)**										
**Modified UT grade (Wagner)**	**0**		**1**		**2**		**3**		**4**	
**UT stage**	**SHC**	**C**	**SHC**	**C**	**SHC**	**C**	**SHC**	**C**	**SHC**	**C**
A n	17	31	8							
%	2.5	6.7	1.2							
B n			10	4		4				
%			1.5	0.9		0.9				
C n	172	75	261	19	38	20	5	18		
%	25.8	16.3	39.1	4.1	5.6	4.3	0.7	3.9		
D n			65	206	56	25	30	32	5	26
%			9.7	44.8	8.4	5.4	4.5	6.9	0.7	5.7
sum n	189	106	344	229	94	49	35	50	5	26
(%)	28.3	23.0	51.5	49.8	13.9	10.6	5.2	10.5	0.7	5.7
**C (2 year follow-up of the disease management program group)**										
**Modified UT grade (Wagner)**	**0**		**1**		**2**		**3**		**4**	
**UT stage**	**SHC**		**SHC**		**SHC**		**SHC**		**SHC**	
A n	58									
%	13.8									
B n			1				1			
%			0.2				0.2			
C n	253		58		10		1			
%	60.4		13.8		2.4		0.2			
D n			13		19		2		3	
%			3.1		4.5		0.5		0.7	
sum	311		72		29		4		3	
(%)	742		17.1		6.9		0.9		0.9	

SHC structured health care program.

Controls.

UT modified University of Texas wound classification system.

The prevalence of wound severity by ulcer grade and stage of both, the patients from the SHC and the control group and the course of the lesions during the two-year follow-up in the group of patients in the SHC program are outlined in Table 3 at admission into the SHC program, about 88% of all lesions were found to penetrate to tendon, capsule, bone or joint or had a local necrosis (modified UT-Wagner grade 2, 3 and 4) and were infected and ischemic (UT- stage D).

At discharge about 30% of all foot wounds were healed. The grade/stage OC signifies healing with continuing compensated ischemia. Another 52% of foot wounds were improved to modified UT-Wagner grade 1.

At the 2 year follow–up examination 74% of the ulcers were healed completely and another 17% were in UT-Wagner grade 1.

Therefore, during the two-year follow-up more than 90% of the ulcers in the patients from the structured health care program were either healed completely or significantly improved.

19.6% of the initial patient group suffered from an ulcer relapse at any point of the study, representing almost the entirety of wounds not healed.

At admission to the clinic about 79% of all lesions in the control group were found to be in modified UT-Wagner grade 2, 3 or 4 and in UT stage D.

At discharge from the clinic 23.0% of all foot wounds of the controls were healed and 49.8% were in modified UT-Wagner grade 1. These subjects had no follow-up control.

The comparison of ulcer healing rates according to the modified UT wound classification system is demonstrated at Figure 2.

The diabetic foot subjects included into the structured health care program had a comparable severity of the lesions at admission to the hospital as compared to the controls.

The average severity of diabetic foot wounds at discharge from the clinic between the treatment groups was tested after adjustment by age, ulcer severity (modified UT classification at admission), PAD, history of CHD, hypertension, smoking and MA. The structured health care group had a significantly (p=0.001) lower level of ulcer severity at discharge compared to controls (Figure 2).

#### Amputations

32 subjects with diabetic foot in the structured health care group underwent major amputation (amputation above the ankle) during hospital treatment (major amputation rate 4.7%). During the two year follow-up 22 subjects underwent major amputation (major amputation rate during follow-up 3.2%).

The major amputations were concentrated in the age group > 65 years.

A relation between the number of major amputations and the grade and stage of the foot lesion could not be established due to the low number of major amputations.

215 out of 684 patients (31.4%) experienced minor amputations (distal of the ankle); the rate of major/minor amputations was about 1:7.

Controls underwent major amputation in 110 cases (21.7%, p< 0.0001 (age adjusted) compared to structured health care group). 179 control patients had minor amputations (35.2%); accordingly, the ratio of major/minor amputations was 1:1.6.

## Discussion

Data depicting the outcome of different treatment regimes for the diabetic foot are heterogeneous throughout literature. Currently, prospective long-term studies regarding diabetic foot management are almost scarce. We therefore conducted this prospective observational study with an 8 year inclusion and a two-year post-treatment observation period. The central objective of the project, a significant reduction of major amputation in diabetic foot patients, has been achieved by introducing a structured health care program for the diabetic foot. The number of amputations above the ankle in this study was 4.7% at the end of the acute hospital treatment period and, thus, very low, in comparison to the control group, treated by usual care of diabetic foot.

This is comparable to the data published by Canavan et al. [11] from the South Tees region in England, demonstrating that an intensified care for patients with diabetic foot results in a drastic reduction of lower extremity amputation (LEA) rates. In this study, the relative risk of a person with diabetes undergoing LEA declined from 46 times of that of a person without diabetes to 7.7 at the end of a 5 year follow up period from 1995 to 2000 and comparable data were published by Krishnan et al. [12].

In a prospective study of a cohort of 291 patients hospitalized for diabetic foot infection at 38 French centres, a LEA rate of 35% during hospital treatment was documented. During the 1 year follow – up LEA rate increased to 48% [13].

The results of centers specialized in the treatment of patients with diabetic foot in Great Britain (Manchester) and the US (San Antonio) were reported by Oyibo et al. [14]. In 1998 and 1999, this observational study comprised 194 patients. The wound data were classified according to the original UT-System.

Only 26% of the ulcers were described to be neuroischemic. The great majority of findings (67%) were neuropathic ulcers. According to these data, a high healing rate should have been expected. However, 14% of the patients underwent LEA due to non-healing ulcers. Four percent of the patients died, and 16% had persistent ulcers at the end of the study. Altogether, 65% of the initially existing ulcers healed completely. The likelihood of calf amputations was 15 times higher for patients with diabetic foot affected by ischemia or infection compared to patients without these conditions.

These data are comparable well to our results presented here especially because the UT system or the modified UT system was used in these studies.

In the EURODIALE-Study, conducted at 14 hospitals in 10 European countries, a major amputation rate of 5.1% in 1229 patients was documented [15].

Severe limb ischemia, as defined by an ABI of <0.5, was present in 12% of these patients. In our study decompensated perfusion was found in 22% of all patients in the disease management program group.

Peripheral vascular disease (PAD) is significantly associated with reduced survival in foot ulcer patients.

Faglia et al. [16] addressed the problem of occlusive peripheral arterial disease in subjects with diabetic foot ulcers. From 1993 to 1995, 121 patients with diabetic foot were admitted consecutively to Milan University hospital. Angiography was carried out in 104 subjects. The most interesting data in this study was the extraordinary high rate of occlusive arterial disease. Only one out of 104 subjects did not have hemodynamically significant stenoses. Nearly half of the patients had stenoses in the popliteal and infrapopliteal axis only. Because neuropathy was also found to be very common too (90 subjects = 86.5%), the prevalent picture was the neuroischemic foot. Similary, more than 80% of all subjects in our study had neuroischemic diabetic foot. In 2009, the same group reported even more impressive data on 554 patients with critical limb ischemia (CLI) [17].

In these patients, peripheral angioplasty (PTA) was performed in 75% and bypass graft (BPG) in 21%. Neither PTA nor BPG were possible in 5% of the subjects.

LEA rate in this highly complicated group of patients was 13.4% (8% in PTA patients, 21% BPG and 59% in the subgroup that received no revascularisation). Comparable data were reported by Uccioli et al. [18]. It can be concluded from these studies that the degree of PAD has a drastic effect on the outcome of diabetic foot therefore limiting the comparability of most of these studies performed at different centers.

The long-term outcome in terms of amputations and mortality in patients with new-onset diabetic foot ulcers in subgroups stratified by etiology was examined by Moulik et al. [19]. Five-year mortality was 18%, 45% and 55% for neuropathic, neuroischemic and ischemic ulcers, respectively.

The rates of mortality also vary significantly between different studies, most likely due to differences in the proportion and degree of CLI, history of foot ulcer or amputation and impaired renal function.

The Milan group [20] evaluated new ulceration, new major amputation and survival rates of 115 subjects with diabetes hospitalized for foot ulceration from 1990 to 1993. One of the main results was the mortality rate of 44% during the follow-up period of 6.5 years. After two years, the mortality rate was around 15% regarding subjects without and more than 40% in subjects with major amputation. The overall mortality rate was 20% after two years. These data are comparable with the mortality rates reported in our present study.

Holstein and Sorensen [21] reported a retrospective study of 162 patients with diabetes with foot ulcers admitted to a vascular surgical department with a new multidisciplinary diabetic foot unit.

The survival rate after 24 months was 68% in subjects with diabetic foot due to peripheral neuropathy without the need for arterial reconstruction, 64% in patients with limb-threatening ischemia undergoing revascularization, and 16% in diabetic foot with limb-threatening ischemia without possible option for revascularization.

Williams et al. [22] recently have shown that the implementation of a multidisciplinary diabetic foot team into a department of vascular surgery was associated with improved outcomes for patients with diabetic foot. The improvements were not related to increased numbers of vascular procedures or hospitalizations, but coincide with greater proportion of patients attending this foot unit.

There is considerable variability in the reported incidence rates of amputation among different countries and various points in time [23-27]. In general a significant reduction in LEA in diabetic foot appears to be realized [28,29]. As compared to the data reported in the literature with major amputation rates of 8-40%, the rate of 4.7% of major amputation in our patients treated by the structured health care program documents a significant improvement.

Armstrong et al. [2] reported that the frequency of major amputation increases with advanced wound progression as determined by the Wagner classification of lesions or with an infection (stage B according to UT system), or ischemia (stage C). A simultaneous occurrence of infection and ischemia (stage D) would further increase the likelihood for LEA.

Thus, if the wound extends to the bone and an infection and ischemia exist (Wagner grade 3 and higher) it is very likely that LEA will be necessary. However, due to the very small number of amputations, the data of our study do not confirm this.

The significantly higher age of the controls in our study may potentially affect wound healing, mortality and amputation rate. Therefore, the data were adjusted to age, PAD, history of CHD, hypertension, smoking and MA.

Moreover, as compared to the controls, the state of perfusion was significantly worse in the patients treated in the structured health care program. Therefore, the significantly higher rate of amputations (4.6-fold) in the control group of our study may not be related to age. Similarly, the increased mortality rate in the controls (3.8-fold) may not be attributed to age differences since both groups (controls/structured health care) did not differ with respect to their general and disease-related morbidity (Table 1).

Patients with diabetic complications and diabetic foot problems in particular are among the most complex and vulnerable of all patient populations and intensive effort is required in these patients in order to accomplish limb preservation [30,31]. The implementation of the structured health care program by the means of a multidisciplinary diabetic foot team is essential to reduce LEA successful [28,32].

The structured health care program for diabetic foot introduced here includes structured outpatient, inpatient and rehabilitative treatment. Since the major amputation rate of the control group without the structured health care program was about 5 times higher, we conclude that a the introduction of a structured health care program can significantly reduce the number of major amputations in patients with diabetic foot.

## Competing interests

The authors declare that they have no competing interests.

## Authors’ contributions

M W researched data, analysed data, and wrote the manuscript. T S, H P, D M, T N, G vG, A B and U D researched data, S B, A B and M H contributed to discussion and reviewed and edited the manuscript. C K had full access to all the data in the study and takes responsibility for the accuracy of data analysis. All authors read and approved the final manuscript.
